# Bibliographical

**Published:** 1880-07

**Authors:** 


### BIBLIOGRAPHICAL.

Outlines of the Practice of Medicine, with a Special Reference to the Prognosis and Treatment of Disease, with Appropriate Formulæ and Illustrations. By Samuel Finwick, M. D., Lecturer on the Principles and Practice of Medicine at the London Hospital, &c.

This is an admirable little compendium of medical practice, prepared by one eminently qualified for the work, being familiar with the practice of medicine, and a teacher withal, so that he is able to concisely prepare and give to others that which he has found most serviceable.

This will be of great value to those who have not the time or opportunity to consult larger and more extended works.

This work will be especially useful to the dentist as a hand-book, which he may consult for many affections that lie in his way, and must receive his attention either in the way of treatment or advice. It is published by Lindsay & Blakiston, and is for sale by Robt. Clarke & Co., of this city. Price $2.

